# Epidemiology and genetic characterization of porcine reproductive and respiratory syndrome virus in Fujian Province, China, from 2023 to 2024

**DOI:** 10.3389/fvets.2025.1634353

**Published:** 2025-07-02

**Authors:** Long-Bin Kang, Qiu-Yong Chen, Bing He, Ren-Jie Wu, Jing-Li Qiu, Ru-Jing Chen, Xue-Min Wu, Long-Bai Wang, Lun-Jiang Zhou

**Affiliations:** ^1^Institute of Animal Husbandry and Veterinary Medicine, Fujian Academy of Agriculture Sciences, Fuzhou, China; ^2^Fujian Animal Disease Control Technology Development Center, Fuzhou, China

**Keywords:** PRRSV, prevalence, gene evolution, Nsp2, ORF5, ORF7

## Abstract

Porcine Reproductive and Respiratory Syndrome (PRRS) is associated with reproductive disorders, respiratory diseases and slower growth rates. PRRSV mutation and recombination lead to the emergence and spread of novel strains, which brings challenges and complexity to clinical prevention and control. However, the epidemical characterization of PRRSV in Fujian is limited. In this study, 262 suspected PRRSV samples from 87 pig farms in Fujian Province, from 2023 to 2024 were collected to monitor the prevalence of PRRSV. Through RT-PCR detection and sequencing of the Nsp2 hypervariable region, ORF5, and ORF7 genes, an analysis of their genetic variation was conducted. The results revealed that the overall prevalence rate of PRRSV was 16.79% (44/262), PRRSV-1 and PRRSV-2 genotypes were co-prevalent in Fujian. Phylogenetic analysis of ORF5 gene identified 37 PRRSV strains, categorizing 1 as PRRSV-1, 36 as PRRSV-2, including 17 strains of NADC30-like subtype (45.95%), 9 strains of lineage 8 (24.32%), 7 strains of lineage 3 (18.92%), 2 strains of NADC34-like subtype (5.41%), and 1 strain of lineage 5 (2.70%). The main way of amino acid change of GP5 is a mutation, and some strains have a deletion. These changes are mainly observed in T cell, B cell epitope region, signal peptide region, and transmembrane region. The above results indicated that NADC30-like was the dominant circulating strain, followed by the HP-PRRSV strain in the farm. Moreover, genetic evolution analysis of the Nsp2 gene showed that the pattern of amino acid deletion between different lineages no longer seems to be applicable as a molecular marker for each lineage, and genetic diversity and recombination were commonly observed. Noteworthy, the identification of a novel independent subtype from the isolated strains indicates that the ORF7 gene also has genetic evolution, which requires us to pay attention to the genetic relationship of ORF7 between the wild strain and the vaccine strain. This study offers crucial insights into the evolutionary dynamics of PRRSV, thus providing a solid foundation for further research into PRRSV epidemiology and control strategies.

## 1 Introduction

Porcine Reproductive and Respiratory Syndrome (PRRS), an immunosuppressive disease induced by Porcine Reproductive and Respiratory Syndrome Virus (PRRSV), leads to reproductive disorders and respiratory diseases, causing significant economic losses in pig farming (1, 2). Since the PRRSV was firstly discovered in the 1980s, the disease has been widely spread and rapidly mutated worldwide (3). According to the genetic differences in the genome, PRRSV is divided into two types: PRRSV-1 typified by the Lelystad Virus strain isolated in the Netherlands, and the PRRSV-2 exemplified by the VR2332 strain isolated in North America (4). Among them, based on the ORF5 variations, the PRRSV-1 is classified into three subtypes (subtype 1,2,3), while PRRSV-2 is divided into nine lineages and 37 sublineages (5).

In China, the CH-1a strain was the first strain that had been reported in 1996 (6), followed by the isolation of the BJ-4 strain in 1997 (7). Both strains exhibited the closest genetic relationship to North American strains and were categorized as PRRSV-2. Over the subsequent decade, PRRSV strains represented by these two became prevalent in China. In 2006, an outbreak of PRRS occurred in Chinese pig farms, marked by high fever and mortality. The etiological agent was identified as the HP-PRRSV strain. Since then, HP-PRRSV has become a predominant circulating strain, incurring economic losses to the pig industry (8). In 2010, the QYYZ strain was reported in pig farms in Guangdong Province, which had been immunized with modified live virus (MLV) vaccines. This strain represents a recombinant of HP-PRRSV and vaccine strains, has emerged as one of the indigenous strains prevalent in South China (9). In 2013, NADC30-like strains rose to prominence as the circulating strains (10). In 2017, NADC34-like strains were first documented in Northeast China (11), analogous to the IA/2014/NADC34 strain from the USA, these variants exhibit 100aa deletion in the Nsp2, and have shown a rising prevalence in China. Moreover, PRRSV-1 strains have been sporadically reported in recent years (12, 13).

The rapid expansion of pig breeding scale and highly intensive breeding in China, coupled with the use of multiple vaccines in PRRSV-infected pig farms provide opportunities for the genetic diversity of porcine reproductive and respiratory syndrome virus (PRRSV). Additionally, uneven biosafety and management practices further exacerbates this situation. At present, the predominant epidemic genotype of PRRSV-2 in China are lineage 1, 3, 5, and 8. The multi-lineage recombination occurs, and the recombination of lineage 1 and 8 is the most commonly observed (14, 15). The mutation and recombination of PRRSV led to the occurrence and dissemination of novel strains, posing challenges and complexities to the clinical prevention and control efforts.

However, the last update on the PRRSV epidemic in Fujian dates back to 2018 (16). Given that the epidemic dynamics of PRRSV may change over time, this study seeks to refresh prior research and present the most recent date on prevalence and genetic evolution. As is widely recognized in the field, sequencing is a key method for analyzing the genetic diversity of PRRSV. Consequently, samples suspected of PRRSV from 2023 to 2024 were collected, detected, and subsequently sequenced. Then the genetic variation in ORF5, Nsp2, and ORF7 genes were subjected to detailed analyzed. This study can help us to accurately identify these genetic variations related to viral characteristics, so as to provide accurate target information for vaccine development, ensure that the vaccine can effectively cope with the changing virus strains, and better prevent and control the diseases caused by PRRSV.

## 2 Materials and methods

### 2.1 Sample collection and processing

Between 2023 and 2024, a total of 87 farms, including family farms and large-scale farms across 8 cities in Fujian province, volunteered to participate in the study. During this period, 262 samples suspected of being infected with PRRSV were collected from these farms. The samples included 57 serum samples, 47 oral fluid samples, 29 testicular fluid samples, and 129 tissue samples. All the farms provided their consent for sample collection. Tissue homogenates were prepared by mixing organ samples (such as lung, spleen, and inguinal lymph node) with DMEM at a 1:10 ratio. The mixture underwent freeze–thaw cycles and was then centrifuged at 3000 rpm for 5 min. After collecting the supernatant, it was centrifuged again at 8000 rpm for another 5 min. Serum, oral fluid, and testicular fluid samples were used right away for the experiments. All the supernatants and serums were kept at −20°C till the RNA extraction. Sample source information is presented in Supplementary Table S1.

### 2.2 Virus RNA extraction, amplification and sequencing

A volume of 200 μL from each sample was utilized for RNA extraction via the kit instructions (BioFlux, Hangzhou, China). cDNA was synthesized using EasyScript® One-Step gDNA Removal and cDNA Synthesis SuperMix kit (Trans, Beijing, China). Then, the ORF7, ORF5, and Nsp2 hypervariable regions were performed using the 2 × TransStart® FastPfu PCR SuperMix (−dye) kit (Trans, Beijing, China) with primers listed in Table 1. The positive bands were excised from the gel and purified with PureLink™ Quick Gel Extraction Kit (Thermo Fisher Scientific, Shanghai, China). The purified products were then ligated into the pUC57 and transformed into DH5α (Tsingke, Shanghai, China). Positive plasmids were identified, and sequenced by Sangon Biotech (Shanghai, China).

**Table 1:** Primers used in this study.

Primer	Sequences (5′-3′)	Production/bp	Aim
ORF7-F	GCCCCTGCCCAYCACG	637/660 (42)	For PRRSV-1 and PRRSV-2
ORF7-R	TCGCCCTAATTGAATAGGTGA		
ORF5-1F	AAGTTATCTTTGGGAACGTCTC	838 (43)	For PRRSV-1
ORF5-1R	GACACCTTAAGGGCATATATCA		
ORF5-2F	GTTTTAGCCTGTCTTTTTGCC	731 (44)	For PRRSV-2
ORF5-2R	TATATCATCACTGGCGTGTAGG		
NSP2-1F	CCTAGCGTCTGCTTACAGACTACC	2,189 (43)	For PRRSV-1
NSP2-1R	AACGCCCCTGGGACACCACATA		
NSP2-2F	GGACACCTCCTTTGATTGGGATG	676 (30)	For PRRSV-2
NSP2-2R	TGAGTATTTTGGGCGCGTGATCT		

### 2.3 Homology, genetic evolution, and mutation analysis

In this study, we considered the predominant PRRSV genotypes in China and selected three representative strains for each genotype, prioritizing the earliest identified ones. Additionally, the vaccine strains and their parent strains, such as CH-1a, MLV, and VR2322 were also considered. In total, 17 PRRSV strains were chosen as reference strains and detailed information were listed in Supplementary Table S2. All the strains in this study and 17 reference strains were performed for homology and phylogenetic analyses. The study analyzed the nucleotide and amino acid homology, as well as mutations in the ORF5, ORF7, and Nsp2 hypervariable regions of the isolated strain compared to reference strains, using MEGA X software (Tempe, AS, USA). Phylogenetic trees were constructed using the Neighbor-joining method with 1,000 bootstrap replicates in MEGA X and were visualized using Evolview. Additionally, the N-linked glycosylation sites in the GP5 protein were predicted via the NetNGlyc 1.0 Serve [NetNGlyc-1.0—redirect (dtu.dk)] (17).

## 3 Results

### 3.1 PRRSV detection and sequencing results of clinical samples

The detection results of clinical samples revealed a PRRSV positivity rate of 16.79% (44/262). After amplification, sequencing, and excluding the identical sequences, 37 different ORF5 gene sequences, 38 different Nsp2 gene hypervariable region (HVR) sequences, and 37 different ORF7 gene sequences were obtained. Unexpectedly, two Nsp2 sequences (FJLY2312(1) and FJLY2312(2)) were performed in one positive sample. These gene sequences have been submitted to GenBank (PQ465885-PQ465996, Supplementary Table S3).

### 3.2 Homology and phylogenetic analysis of ORF5

**Figure 1:**
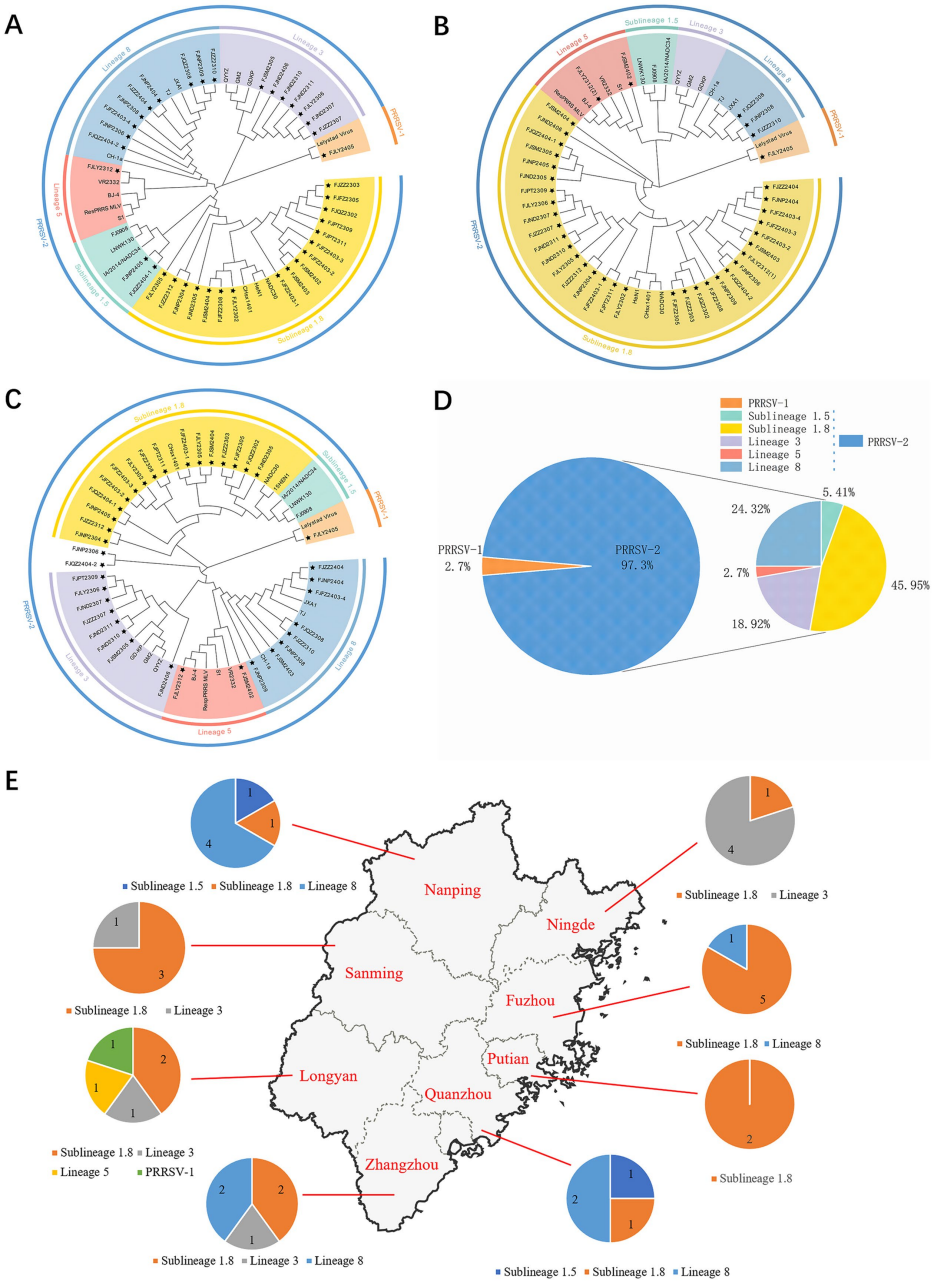
Phylogenetic tree of all PRRSV strains in this study and their geographical distribution. **(A)** ORF5 gene nucleotides; **(B)** Nsp2 gene nucleotides; **(C)** ORF7 gene nucleotides; **(D)** Percentage ratio of each lineage; **(E)** The geographical distribution of different genotypes PRRSV strains within Fujian.

**Figure 2:**
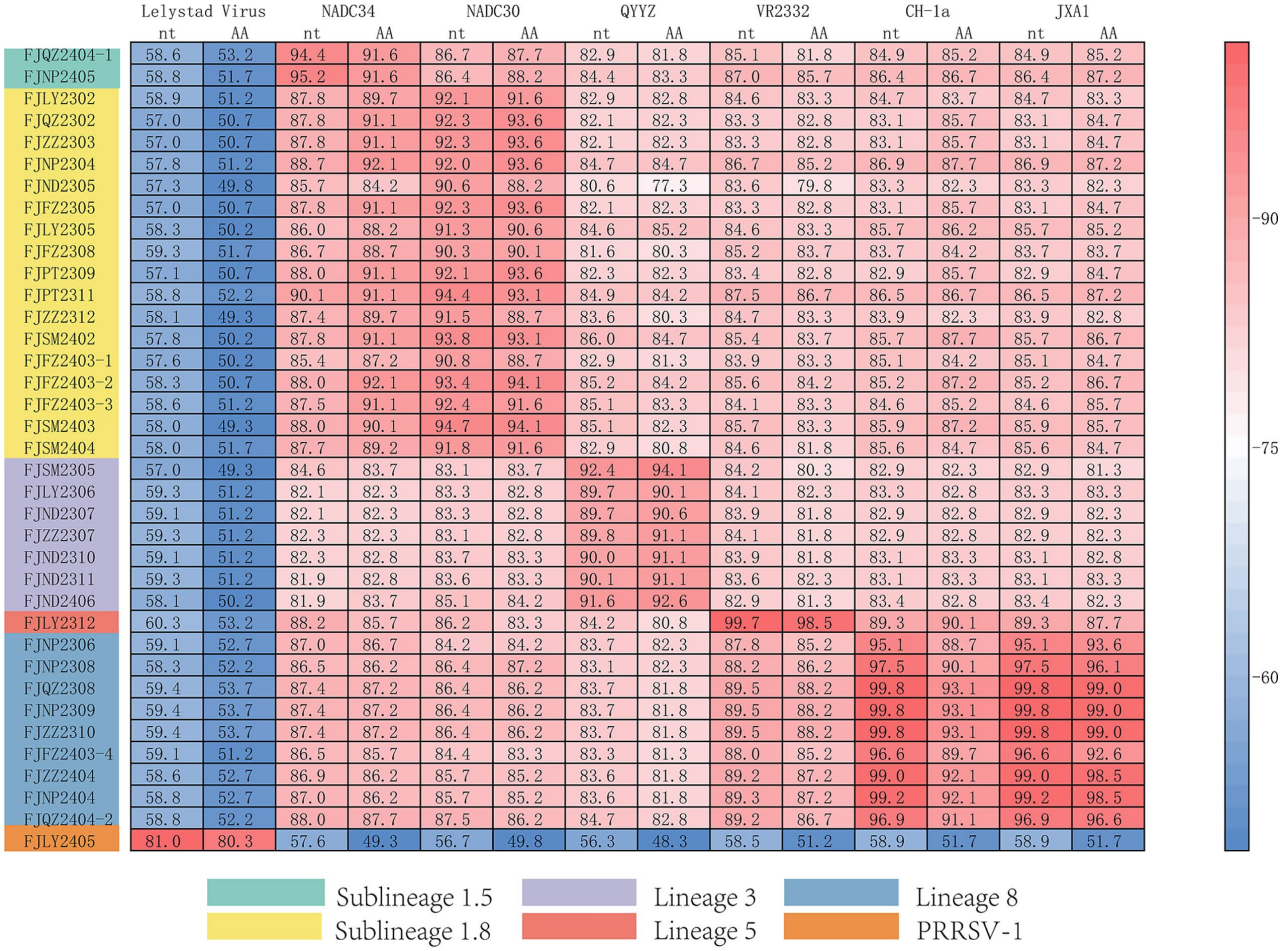
Homology analysis of ORF5 gene.

The ORF5 gene encodes the main viral envelope glycoprotein, which has significant genetic diversity and is considered the basis for PRRSV lineage division. As showed in Figures 1A,D, 2, among the 37 isolated strains, 1 strain belonged to PRRSV-1, and shared the 81.0% nucleotide and 80.3% amino acid homology with the Lelystad virus strain, respectively. The remaining 36 strains belonged to PRRSV-2, and 17 strains were closely related to the lineage 1.8 (NADC30-like) (Figures 1A,D, 2), which had the highest prevalence at 45.95% (17/37), showing 90.6–94.7% nucleotide and 88.2–94.1% amino acid identity with the reference NADC30 strain, respectively. 7 lineage 3 strains accounted for 18.92% (7/37), with 89.7–92.4% nucleotide and 90.1–94.1% amino acid identity with the QYYZ strain. FJQZ2404-1 and FJNP2405 strains were of the lineage 1.5 (NADC34-like), made up a prevalence of 5.41% (2/37) of cases and shared 94.4–95.2% nucleotide and 91.6% amino acid identity with the other NADC34 strains. FJLY2312 strain belonged to lineage 5, showing 99.7% nucleotide and 98.5% amino acid identity with VR2332. 9 strains were closely related to lineage 8, accounted for 24.32% (9/37) of positive samples, with 95.1–99.8% and 95.1–99.8% nucleotide sequence identity with JXA1 and CH-1a, respectively, and deduced amino acid sequence identity of 88.7 ~ 93.1% and 92.6 ~ 99.0%. The geographical distribution of different genotypes PRRSV strains within Fujian was shown in Figure 1E.

### 3.3 Amino acid mutation, functional domain, and N-glycosylation site analysis of GP5

**Figure 3:**
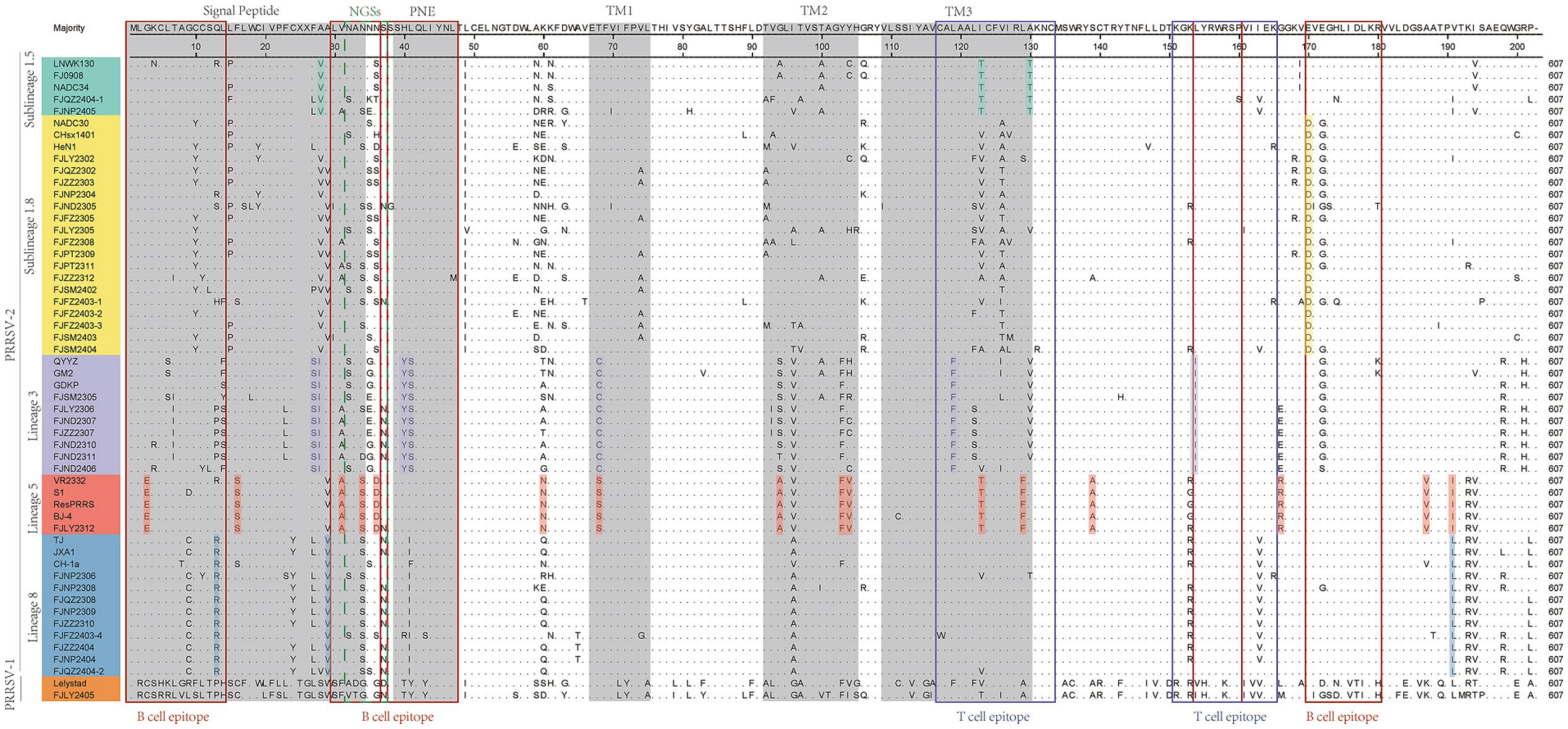
The results of multiple alignment of the GP5 amino acid sequences. B-cell epitopes, T-cell epitopes, and regions of changes in NGSs are indicated by red, black, and dashed boxes, respectively, and the signal peptide and the three transmembrane regions are indicated in gray.

As showed in Figure 3, GP5 proteins of PRRSV-1 and PRRSV-2 comprised 201 and 200 amino acids, respectively. Compared with PRRSV-2, PRRSV-1 strain had one insertion at ^24^TG^25^ and one deletion occurred at the 199th position. The amino acid sequences of 36 PRRSV-2 isolated strains were compared with the reference strains, among them 7 strains had amino acid deletion, FJSM2305 strain had one deletion at N^32^, FJQZ2302, FJZZ2303, FJFZ2305, FZFZ2308, FJPT2309, and FJSM2404 strains had one deletion at N^35^, no other deletion or addition occurred in other region. However, the amino acid substitutions were frequently observed, mainly occurring in T cell and B cell epitopes. Specifically, amino acids mutated in the GP5 protein signal peptide region, such as C^11^ → Y^11^, W^18^ → L^18^, C^19^ → Y^19^, F^23^ → L^23^ or S^23^, L^29^ → I^29^. And amino acid mutations also be observed in the transmembrane region, such as V^70^ → I^70^, V^74^ → A^74^, T^97^ → A^97^ or V^97^, H^105^ → R^105^, S^111^ → R^111^, C^117^ → W^117^, I^127^ → V^127^, M^127^, or L^127^ (Figure 3). The VR2332 strain has been considered to be a classical strain of the PRRSV-2 genotype and is the parental strain of the MLV vaccine, which had been applied in farms of China (3). Compared with VR2332 (Figure 3), the amino acid regions of 76–91,107–118 and 173–186 of 36 PRRSV-2 strains in this study were relatively conserved.

Additionally, there are unique amino acid mutations between different lineages (Supplementary Table S4). Such as, all the sublineage 1.5 strains contained two unique amino acid mutations, T^121^ and T^128^, which are located in the T cell epitopes. In the B cell epitope, the sublineage 1.8 strains contained two unique amino acid mutations, N/S^33^ and D^168^. And the strains belonged to lineage 3 had four amino acid mutations in T cell and B cell epitope, which are ^38^YS^39^, F^117^, and I^152^. Lineage 5 had three amino acid mutations in T cell epitope and B cell epitope, which are E^3^, D^34^, and T^127^. Lineage 8 had one amino acid mutation (R^14^ or I/F^39^) in B cell epitopes. Moreover, within the signal peptide region, sublineage 1.5 is characterized by the specific amino acid site V^26^. For lineage 3, two consecutive sites, 25^S^ and 26^I^ are uniquely associated. In lineage 5, the site S^16^ serves as a lineage-specific marker (Figure 3 and Supplementary Table S4). In the transmembrane region, lineage 3 features the distinct site C^68^. Lineage 5 contains two key site motifs: A^92^ and ^101^FV^102^. Lineage 5-specific residues include N^58^, A^137^, R^164^, and V^185^. Notably, I^189^ is shared as a lineage-defining marker between lineage 5 and lineage 8 (Figure 3 and Supplementary Table S4).

The 13th and 151th amino acids in GP5 protein correlate with viral virulence, and R^13^, R^151^ are the characteristics of virulent strains (18). Among the 37 strains (Figure 3), at the 13th position, 10 strains had R^13^ residue, one strain was mutated with H^13^, one strain was mutated with S^13^ and 6 strains were mutated with P^13^, and the remaining strains were mutated with Q^13^. And at the 151th position, 12 strains were mutated with R^151^, 8 strains were mutated with I^151^, and the remaining strains were mutated by K^151^. In addition, the seven strains (FJNP2404, FJZZ2404, FJFZ2403-4, FJZZ2310, FJNP2309, FJQZ2308, and FJNP2308) shared R^13^ and R^151^ residues (Figure 3), which were consistent with the results of the ORF5 gene phylogenetic tree and belonged to the lineage 8 (Figure 1A).

N^30^, N^32/33/34^, N^35^, N^44^, and N^51^ were GP5 protein N-glycosylation sites, which are closely related to virus infectivity and immune evasion (19). All isolate strains were predicted to have 3–5 NGS, which were conserved at N^44^ and N^51^, while the NGS in front of N^44^ were relatively variable (Table 2). In all strains except FJZZ2312, N-glycosylation sites at N^44^L^45^T^46^ and N^51^G^52^T^53^ were highly conserved, without mutation or deletion. Specifically, FJZZ2312 had a mutation of L^45^ → M^45^ (Figure 3). The QYYZ-like strain FJSM2305 lacked a NGS at N^32^, and six NADC30-like strains (FJQZ2302, FJZZ2303, FJFZ2305, FZFZ2308, FJPT2309, and FJSM2404) lacked a NGS at N^35^, and no other deletion in remain isolates (Table 2).

**Table 2:** Potential N-glycosylation sites (NGS) on GP5 protein.

Isolates	Lineage	N-glycosylation sites							NGSs number
		N30	N32	N33	N34	N35	N44	N51	
NADC34	1.5		√	√			√	√	4
FJQZ2404-1	1.5		√				√	√	3
FJNP2405	1.5	√			√		√	√	4
NADC30	1.8				√		√	√	3
FJLY2302	1.8		√	√		-	√	√	4
FJQZ2302	1.8		√				√	√	3
FJZZ2303	1.8		√			-	√	√	3
FJNP2304	1.8			√	√		√	√	4
FJND2305	1.8	√				√	√	√	4
FJFZ2305	1.8		√			-	√	√	3
FJLY2305	1.8				√		√	√	4
FJFZ2308	1.8		√	√		-	√	√	4
FJPT2309	1.8		√				√	√	3
FJPT2311	1.8			√		-	√	√	3
FJZZ2312	1.8	√		√			√	√	4
FJSM2402	1.8				√		√	√	3
FJFZ2403-1	1.8	√				√	√	√	4
FJFZ2403-2	1.8			√	√		√	√	4
FJFZ2403-3	1.8			√	√		√	√	4
FJSM2403	1.8				√		√	√	3
FJSM2404	1.8		√	√		-	√	√	4
QYYZ	3				√		√	√	3
FJSM2305	3		-		√		√	√	3
FJLY2306	3	√			√	√	√	√	5
FJND2307	3				√	√	√	√	4
FJZZ2307	3				√	√	√	√	4
FJND2310	3				√	√	√	√	4
FJND2311	3				√	√	√	√	4
FJND2406	3				√		√	√	3
VR2332	5	√		√			√	√	4
FJLY2312	5	√				√	√	√	4
TJ	8	√			√	√	√	√	5
JXA1	8	√			√	√	√	√	5
CH-1a	8				√		√	√	3
FJNP2306	8			√	√		√	√	4
FJNP2308	8	√			√	√	√	√	5
FJQZ2308	8	√			√	√	√	√	5
FJNP2309	8	√			√	√	√	√	5
FJZZ2310	8	√			√	√	√	√	5
FJFZ2403-4	8			√			√	√	3
FJZZ2404	8	√			√	√	√	√	5
FJNP2404	8	√			√	√	√	√	5
FJQZ2404-2	8	√			√	√	√	√	5
Lelystad virus	PRRSV-1						√	√	2
FJLY2405	PRRSV-1					√	√	√	3

### 3.4 Nucleotide homology, amino acid mutation and phylogenetic tree analysis of Nsp2 gene

**Figure 4:**
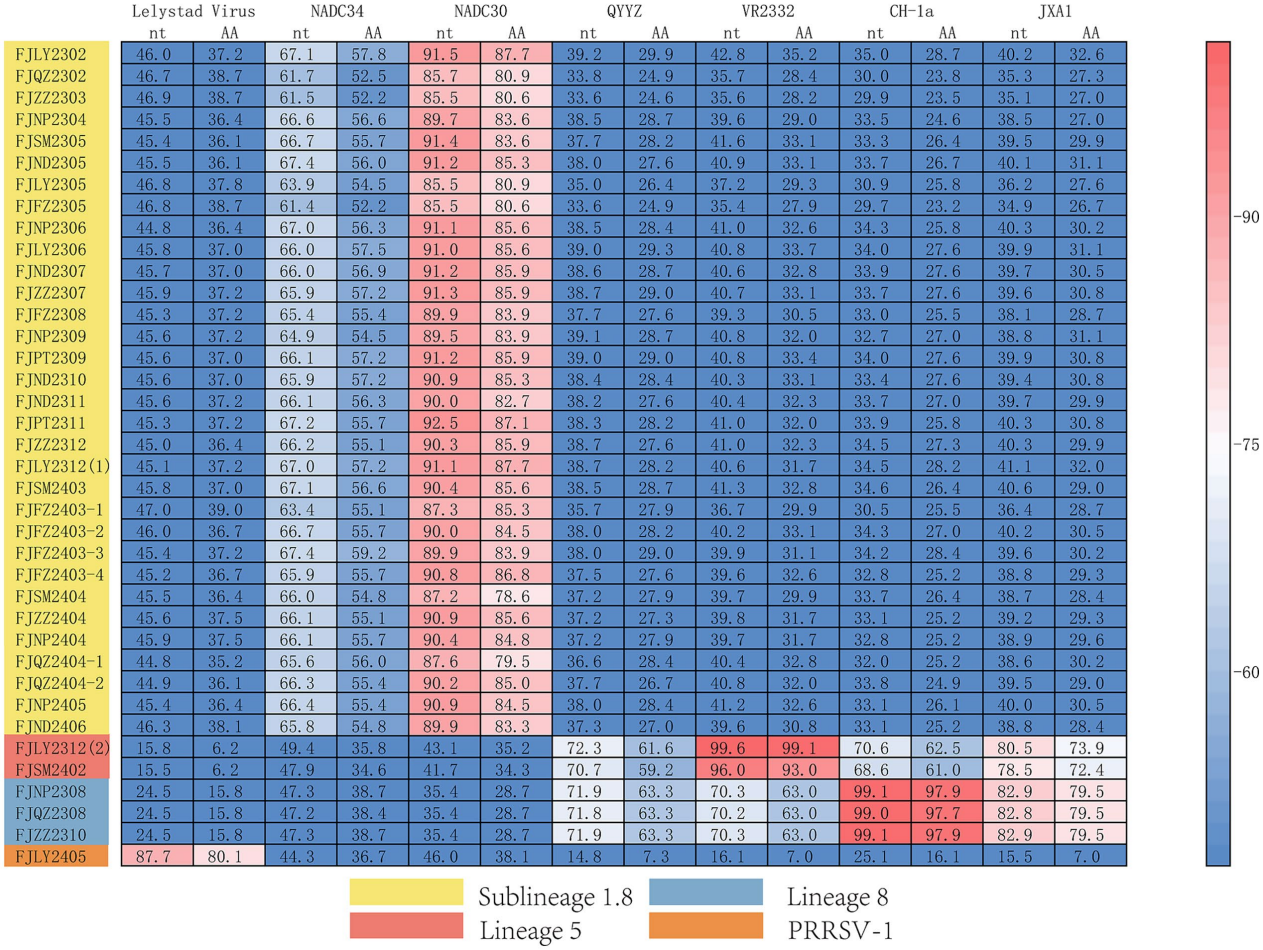
Homology analysis of Nsp2 gene.

The results of nucleotide alignment and homology (Figures 1B, 4) showed that 32 isolated strains belonged to the NADC30-like subtype, and share 85.5 ~ 92.5% homology with the NADC30 reference strain. 2 strains belonged to the lineage 5, and the homology with the VR2332 strain was 96.0 ~ 99.6%. 3 strains belonged to the lineage 8, and the homology with CH-1a and JXA1 strains was 99.0 ~ 99.1% and 97.7 ~ 97.9%, respectively. 1 strain belonged to the PRRSV-1 type, and the homology with Lelystad strains was 87.7%.

**Figure 5:**
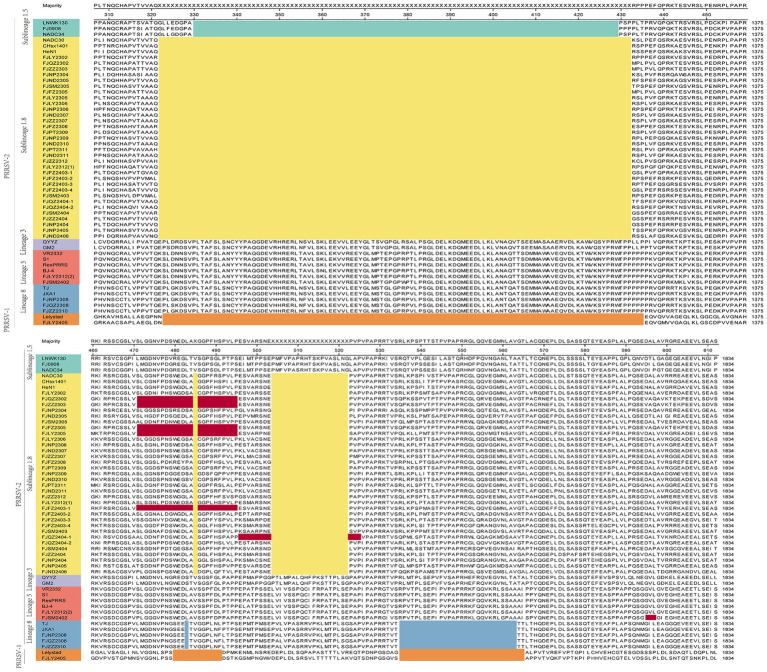
The multiple alignment analysis of highly variable region of Nsp2. Navy blue boxes indicate consecutive 100 aa deletions in NADC34-like strains, yellow boxes indicate discontinuous 131 aa deletions in NADC30-like strains, blue boxes indicate discontinuous 30 deletions in HP-PRRSV strains, orange boxes indicate deletions in PRRSV-1 compared to VR2332, and red boxes indicate additional occurrences of deletions.

Moreover, we also analyzed the 37 PRRSV strains’ amino acid sequences. Practically, 38 Nsp2 gene sequences were multiple alignment because FJLY2312 contained two Nsp2 sequences. The results (Figure 5) showed that compared with the VR2322, the amino acids of FJSM2402 and FJLY2312 (2) strains were the most similar, but the FJSM2402 strain had a unique GVL deletion at the 595-597th position. The 32 PRRSV strains represented by FJLY2302 had a discontinuous deletion of 131aa (111 + 1 + 19aa) in 322aa-432aa, 485aa and 504aa-522aa, which was consistent with the characteristic deletion of NADC30-like subtype. However, 5 out of 32 strains (FJQZ2302, FJZZ2303, FJFZ2305, FJLY2305, and FJFZ2403-1) had an additional 24aa (located at 471aa-484aa and 486aa-495aa) deletion. 1 out of 32 strains (FJQZ2404-1) showed an additional 11aa (located at 496aa-503aa and 523aa-525aa) deletion. Apart from this, 3 strains represented by FJZZ2310 had 30 discontinuous deletions (1 + 29aa), which was consistent with the deletion of HP-PRRSV type. In addition, FJLY2305 isolated strain belongs to PRRSV-1, and there are more deletions in the Nsp2 than in PRRSV-2.

### 3.5 Nucleotide homology, amino acid mutation and phylogenetic tree analysis of ORF7 gene

**Figure 6:**
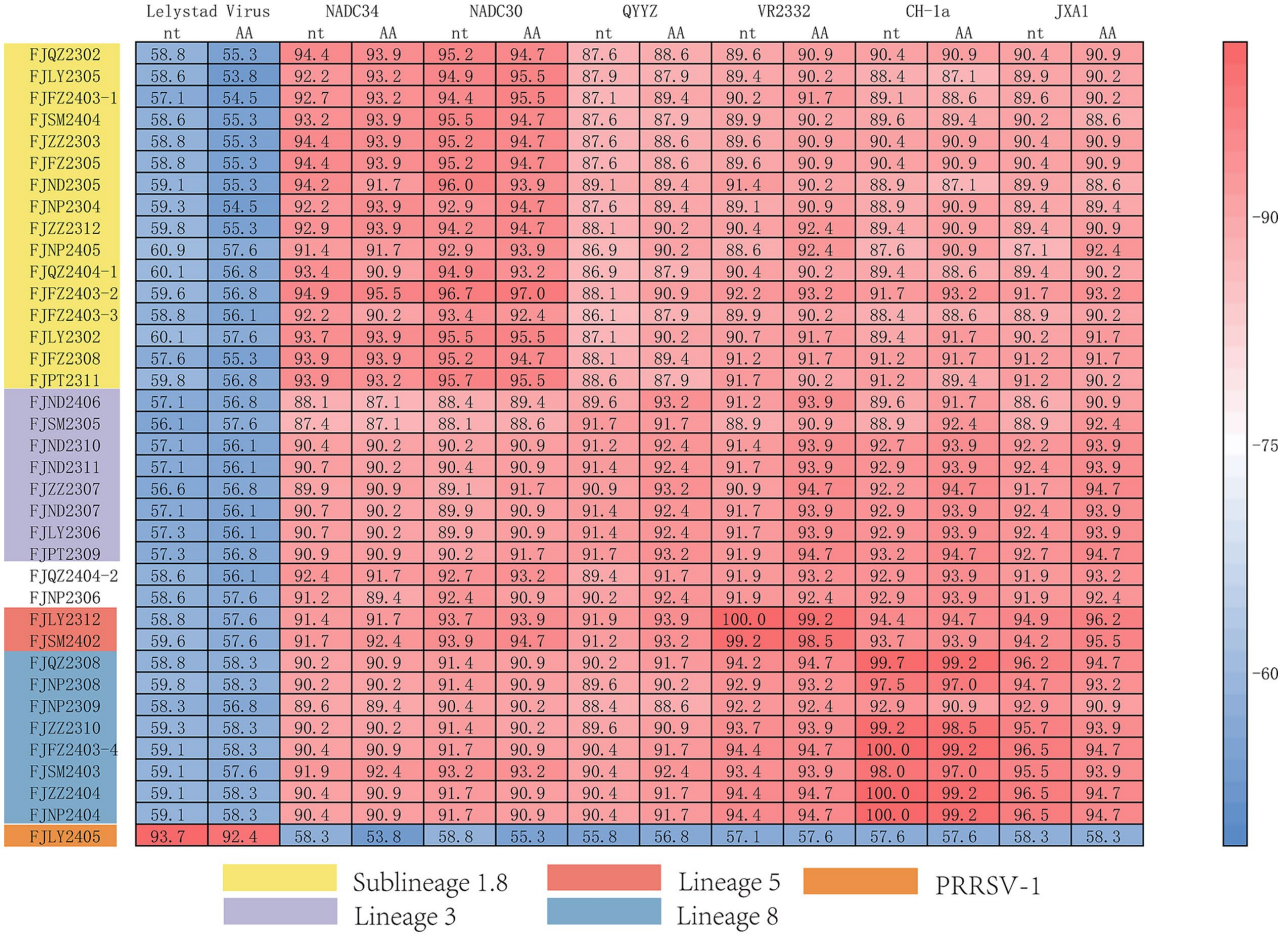
The multiple alignment analysis of ORF7.

The nucleotide alignment results (Figures 1C, 6) showed that 8 PRRSV strains belonged to lineage 8, HP-PRRSV-like subtype, and shared 92.9 ~ 100% diversity with reference strains. Notably, 2 strains exhibited a close genetic relationship with Lineage 5 (VR2332-like), displaying a homology of 99.2 ~ 100% compared to the reference strains. Additionally, 8 strains were classified as QYYZ-like of Lineage 3, with a homology range of 89.6 ~ 91.7% relative to the reference strains. Furthermore, a significant proportion of the strains, totaling 16, were identified as NADC30-like of Lineage 1.8, and these strains showed a considerable homology of 92.9 ~ 96.7% with the representative strains. However, 2 PRRSV strains (FJQZ2404-2 and FJNP2306) showed low similarity with the all-representative strains and formed a novel sublineage (Figure 1C). Their homology with the reference strains of NADC34, NADC30, QYYZ, VR2332, CH-1a, and JXA1 were 91.2 ~ 92.4%, 92.4 ~ 92.7%, 89.4 ~ 90.2, 91.9, 92.9, and 91.9%, respectively. In the phylogenetic tree based on ORF5, these 2 strains (FJQZ2404-2 and FJNP2306) were located in lineage 8, while they were belonged to NADC30-like sublineage based on Nsp2 phylogenetic tree. This indicates that there is genetic diversity among the different genes of these 2 strains.

**Figure 7:**
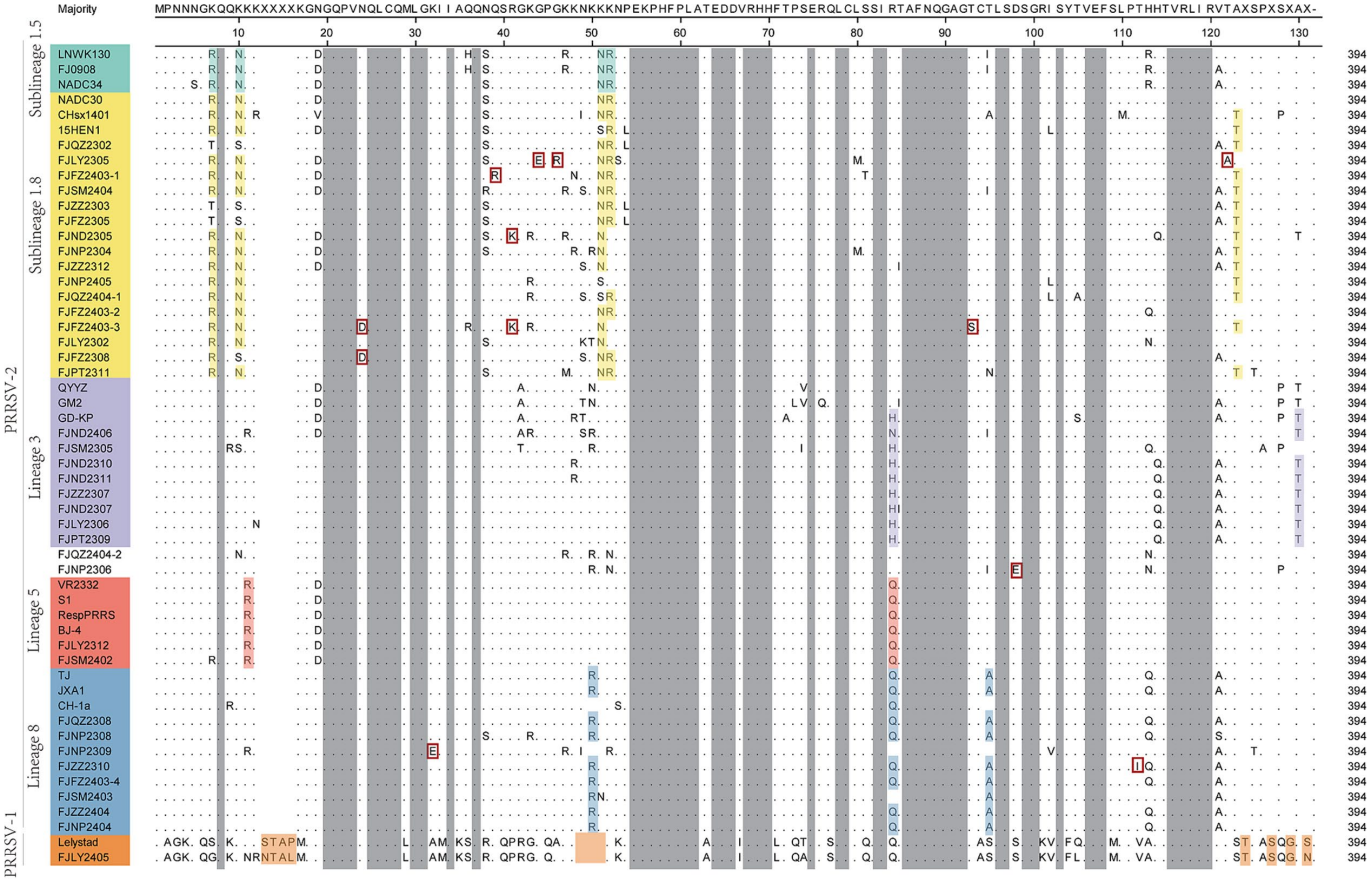
The multiple alignment analysis of ORF7 gene.

In the analysis of amino acid changes, the FJLY2405 strain (belonged to PRRSV-1) exhibited relatively low homology of 55.8% ~ 58.8% with PRRSV-2 strains (Figure 6). The comparison of deduced amino acid sequences revealed numerous insertions, deletions, and substitutions. Besides, variations also existed among different lineages of PRRSV-2. As shown in Figure 7, lineage 1 strains showed a preference for R^7^, N^10^, N^47^, R^48^, and T^120^. Lineage 3 strains favored H^80^ and T^124^. Lineage 5 strains favored R^11^ and Q^80^. Lineage 8 strains favored R^46^ and Q^80^. In addition, several mutations were observed in some isolated strains, such as 20^N^ → 20^D^ in FJFZ2308 and FJFZ2403-3, 28^K^ → 28^E^ in FJNP2309, 35^Q^ → 35^R^ in FJFZ2403-1, 37^R^ → 37^K^ in FJND2305 and FJFZ2403-3, 40^G^ → 40^B^ and 42^G^ and 42^R^ in FJLY2305, 89^T^ → 89^S^ in FJFZ2403-3, 94^D^ → 94^E^ in FJNP2306, 108^T^ → 108^I^ in FJZZ2310,and 118^T^ → 118^S^. These mutations may represent potential mutation sites in the N protein.

## 4 Discussion

The ongoing recombination of PRRSV has significantly boosted its genetic diversity in China, thereby complicating and challenging the disease’s prevention and control (20). As reported by Li et al. (21), from 2021 to 2022, the overall positive rate of PRRSV in China was 23.7%. Notably, the northwest region had an exceptionally high infection rate of 84.6%, whereas the southwest region recorded a 48.2% infection rate. Mei et al. (22) indicated that between 2022 and 2023, the prevalence of PRRSV in Jiangsu, Anhui, and Zhejiang was 13.7, 11.9, and 12.0%, respectively, with NADC34-like and HP-PRRSV being the predominant lineages. Na et al. (23) highlighted that from 2021 to 2023, the positive rates in Hebei, Shanxi, and Liaoning were 13.20, 21.08, and 21.88%, respectively. The dominant strain was NADC30-like, followed by NADC34-like and HP-PRRSV, reflecting regional variations in infection rates. In our study, analysis of 262 samples collected across Fujian province revealed a PRRSV positive rate of 16.79%. Phylogenetic analysis based on ORF5 sequences demonstrated the PRRSV-1 and PRRSV-2 were circulating in Fujian province. Furthermore, within the PRRSV-2 group, virus belonged to the NADC30-like subtype was predominant circulating strain. In recent years, NADC34-like strains have been isolated and identified, and are reportedly becoming predominant circulating strain (11, 24). However, in this study, NADC30-like strains were still the main ones detected in positive samples. This might be due to the limited number of collected samples. Additionally, the implementation of biosecurity measures for African swine fever prevention and control may have decreased the introduction of exogenous PRRSV. Moreover, some pig farms in Fujian did not use PRRSV vaccines, which may reduce PRRSV recombination. Therefore, further research is needed to monitor NADC34-like strains in the future.

The GP5 protein of PRRSV exhibits significant genetic variability, being one of the most variable regions among the virus’s structural proteins. It contains a site that determines viral virulence and is crucial for inducing immune responses (25). In this study, the amino acid changes in GP5 were primarily mutations, with some strains showing deletions. These mutations were mainly displayed in T cell and B cell epitopes, also occurred in transmembrane and signal peptide regions, aligning with previous reports (20, 26). Compared to classical vaccine strains like VR2332 or CH-1a, the NADC30-like strain isolated in this study displayed unique amino acid variations at positions T^121^ and T^128^, while the NADC34-like isolates had unique variations of N/S^33^ and D^168^, located on T cell and B cell epitopes, respectively. These variations might enable the virus to escape neutralizing antibody recognition and could be a key factor in vaccine immunization failure (27). The 13th and 151th amino acids of the GP5 protein are associated with virulence, previous studies have documented that in most NADC34-like and NADC30-like strains, the 13th amino acid mutated from R to Q, and the 151th from R to K (26). In this study, besides the Q mutation at position 13, PRRSV isolates also exhibited other mutations with P, H, and S. At position 151, in addition to K, mutations to I were observed. These findings highlight the diversity of GP5 mutations in NADC30-like and NADC34-like strains. Such mutations may drive strain development toward lower virulence (18), potentially explaining the previously reported the pathogenicity of NADC30-like and NADC34-like strains was inconsistent within lineages and between lineages (28, 29). In the future, it is necessary to further study the relationship between different amino acid mutations and the virulence of strains. Changes in the N-glycosylation site of PRRSV are associated with viral replication, immune escape, and induction of immune responses (30). Deleting N^35^ can hinder PRRSV replication *in vivo* but also prompt faster production of neutralizing antibodies (31, 32). In this study, six NADC30-like strains lacked the N-glycosylation site at N^35^. This deletion might lead to asymptomatic infections in pigs and could underpin the widespread prevalence of NADC30-like strains on pig farms.

Nsp2 is the region with the highly genetic variation in Nsp genes, which is related to the genetic evolution of virus (33). Previously, the Nsp2 of NADC34-like strain was reported to have a continuous 100 amino acid deletion (34). In this study, we found that FJQZ2404-1 and FJNP2405 strains that belong to the NADC34-like based on the ORF5 phylogenetic tree, did not have typical deletion pattern in Nsp2 gene. In addition, Nsp2 gene was no continuous 30aa deletion feature of HP-PRRSV in six strains (FJNP2309, FJNP2404, FJZZ2404, FJFZ2403-4, FJNP2306, and FJQZ2404-2), which belonged to HP-PRRSV in lineage8 based on the ORF5 phylogenetic tree. These findings further reinforce the conclusion that the 30aa deletion in the Nsp2 coding region can no longer be regarded as a definitive molecular marker for HP-PRRSV in China (35). Similarly, the seven strains in PRRSV-2 lineage3 based on the ORF5 phylogenetic tree did not have 36 aa insertions but had 111 + 19 + 1 amino acid deletion, which was the same as the typical NADC30 deletion pattern. Unexpectedly, we amplified two NSP2 sequences in the FJLY2312 strain, which were consistent with the deletion patterns of NADC30 and VR2322, respectively. This result further indicates that the live vaccine strain is recombined with the wild strain in the pig farm. The above results indicate that the genetic diversity among different genes of PRRSV, and the existence of wild virus and vaccine recombination, the coexistence of multiple strains in a pig farm, poses difficulties for the prevention and control of PRRSV.

ORF7 is regarded as a target gene for PCR detection and a target protein for ELISA kits because of its conservative gene sequence and its immunogenic epitopes (36). However, N protein is also used to monitor the genetic variation and genetic evolution of PRRSV (37). Hao et al. reported that ORF7 can be divided into five subtypes, and HP-PRRSV strains are distantly related to MLV or CH-1a vaccine strains, which may affect the immune effect of vaccines (38). In this study, in addition to belonging to HP-PRRSV, VR2322, QYYZ, NADC30, and NADC34 strains, there are two strains (FJQZ2404-2, FJNP2306) that form an independent branch and display 91.9%, or 92.9% amino acid homology with MLV or CH-1a vaccine strains, means that PRRSV in the epidemic process of N protein also exist genetic variation. Further, based on ORF5 phylogenetic analyses, these two strains were classified into the HP-PRRSV subgroup, while the phylogenetic tree of Nsp2 gene showed that they belonged to the NADC30-like subgroup, indicating that there was genetic diversity between different ORFs. A potential account for the clumped patterns observed across various ORF phylogenetic trees is genetic recombination (39). In the study by Tian et al. (40), the phylogenetic analysis of Nsp2 classified PRRSV BX/CH/22 exhibits similarity to the JXA1-type PRRSV, and ORF5 analysis classified it as NADC 30-like PPRSV, then recombination analysis revealed that BX/CH/22 contains an NADC 34-like PRRSV backbone, an Nsp-coding region from NADC 30-like PRRSV. Jeong et al. (41) reported that the phylogenetic and recombination analyses are consistent, as the major parental regions of GNU-2353 Nsp2 cluster with NADC34, while the minor parental regions of both GNU-2353 and GNU-2377 strains correlate closely with those of RespPRRS MLV. So, when the PRRSV genome cannot be obtained, the PRRSV recombination may be predicted by sequencing ORF5, ORF7, and Nsp2, especially in large-scale investigations.

Therefore, predicting the pathogenicity of PRRSV and selecting vaccines based only on the sequencing data of the ORF5 and Nsp2 genes is limited. We should also detect the genetic evolution of the ORF7 gene. In addition, we need to consider the diversity of PRRSV strains, vaccination, and health management strategies to guide the clinical prevention and control of PRRSV. However, a potential limitation is that the representativeness of the sample might be constrained. This is because voluntary participating pig farms could share certain common characteristics. Moreover, there may be differences between these voluntary participating farms and those that do not participate voluntarily in terms of PRRSV infection and other factors. Such differences might impact the broad applicability of the research findings. Further research with larger sample sizes is needed to gain a more comprehensive understanding of the genetic diversity of PRRSV.

## 5 Conclusion

The NADC30-like was the dominant circulating strain of PRRSV in Fujian province, followed by HP-PRRSV strain. The ORF5, Nsp2, and ORF7 genes exhibit genetic diversity and have distinct topological evolutionary relationships. These findings shed new light on the prevalence of PRRSV in Fujian. Considering the genetic diversity of PRRSV and the efficacy of vaccines, it is essential to continuously monitor PRRSV and enhance disease prevention, control, and risk warning measures accordingly.

## Data Availability

The datasets presented in this study can be found in online repositories. The names of the repository/repositories and accession number(s) can be found in the article/Supplementary material.

## References

[1] Wang G Yu Y He X Wang M Cai X Zimmerman JJ. Porcine reproductive and respiratory syndrome virus infection of bone marrow: lesions and pathogenesis. Virus Res. (2019) 265:20–9. doi: 10.1016/j.virusres.2019.02.019, PMID: 30831176

[2] Fang K Liu S Li X Chen H Qian P. Epidemiological and genetic characteristics of porcine reproductive and respiratory syndrome virus in South China between 2017 and 2021. Front Vet Sci. (2022) 9:853044. doi: 10.3389/fvets.2022.853044, PMID: 35464348 PMC9024240

[3] He Z Li F Liu M Liao J Guo C. Porcine reproductive and respiratory syndrome virus: challenges and advances in vaccine development. Vaccines. (2025) 13:260. doi: 10.3390/vaccines13030260, PMID: 40266104 PMC11945896

[4] Brinton MA Gulyaeva AA Balasuriya UBR Dunowska M Faaberg KS Goldberg T. Ictv virus taxonomy profile: Arteriviridae 2021. J Gen Virol. (2021) 102:1632. doi: 10.1099/jgv.0.001632, PMID: 34356005 PMC8513641

[5] Zhou L Han J Yang H. The evolution and diversity of porcine reproductive and respiratory syndrome virus in China. Vet Microbiol. (2024) 298:110252. doi: 10.1016/j.vetmic.2024.110252, PMID: 39299012

[6] Guo BQ Chen ZS Liu WX Cui YZ. Porcine reproductive and respiratory syndrome virus was isolated from abortive fetus of suspected Prrs. Chin J Anim Poult Infect Dis. (1996) 87:1–5.

[7] Yang H Guan S Yin X Gan M. Isolation and preliminary identification of porcine reproductive and respiratory syndrome virus. Chin J Vet Med. (1997) 10:9–10.

[8] Tian K Yu X Zhao T Feng Y Cao Z Wang C. Emergence of fatal Prrsv variants: unparalleled outbreaks of atypical Prrs in China and molecular dissection of the unique Hallmark. PLoS One. (2007) 2:e526. doi: 10.1371/journal.pone.0000526, PMID: 17565379 PMC1885284

[9] Su Z Wang X Liu K Chen G Zhang K Liu J. Recombination and pathogenicity analysis of Nadc30-like and Qyyz-like Prrsv strains in South China. Microb Pathog. (2025) 200:107351. doi: 10.1016/j.micpath.2025.107351, PMID: 39890085

[10] Zhang Q Jiang P Song Z Lv L Li L Bai J. Pathogenicity and antigenicity of a novel Nadc30-like strain of porcine reproductive and respiratory syndrome virus emerged in China. Vet Microbiol. (2016) 197:93–101. doi: 10.1016/j.vetmic.2016.11.010, PMID: 27938690

[11] Yuan L Zhu Z Fan J Liu P Li Y Li Q. High pathogenicity of a Chinese Nadc34-like Prrsv on pigs. Microbiol Spectr. (2022) 10:e0154122. doi: 10.1128/spectrum.01541-22, PMID: 35766496 PMC9431460

[12] Huang B Xu T Luo Z Deng L Jian Z Lai S. Prevalence and genetic diversity of Prrsv in Sichuan Province of China from 2021 to 2023: evidence of an ongoing epidemic transition. Virology. (2024) 600:110213. doi: 10.1016/j.virol.2024.110213, PMID: 39265448

[13] Guo Z Chen XX Li R Qiao S Zhang G. The prevalent status and genetic diversity of porcine reproductive and respiratory syndrome virus in China: a molecular epidemiological perspective. Virol J. (2018) 15:2. doi: 10.1186/s12985-017-0910-6, PMID: 29301547 PMC5753475

[14] Luo Q Zheng Y He Y Li G Zhang H Sha H. Genetic variation and recombination analysis of the Gp5 (Gp5a) gene of Prrsv-2 strains in China from 1996 to 2022. Front Microbiol. (2023) 14:1238766. doi: 10.3389/fmicb.2023.1238766, PMID: 37675419 PMC10477998

[15] An TQ Tian ZJ Leng CL Peng JM Tong GZ. Highly pathogenic porcine reproductive and respiratory syndrome virus, Asia. Emerg Infect Dis. (2011) 17:1782–4. doi: 10.3201/eid1709.110411, PMID: 21888830 PMC3322091

[16] Xie S Liang W Wang X Chen H Fan J Song W. Epidemiological and genetic characteristics of porcine reproduction and respiratory syndrome virus 2 in mainland China, 2017-2018. Arch Virol. (2020) 165:1621–32. doi: 10.1007/s00705-020-04661-z, PMID: 32409873

[17] Gupta R Brunak S. Prediction of glycosylation across the human proteome and the correlation to protein function. Pac Symp Biocomput. (2002) 7:310–22. PMID: 11928486

[18] Li P Shen Y Wang T Li J Li Y Zhao Y. Epidemiological survey of Prrs and genetic variation analysis of the Orf5 gene in Shandong Province, 2020-2021. Front Vet Sci. (2022) 9:987667. doi: 10.3389/fvets.2022.987667, PMID: 36187820 PMC9521713

[19] Akter F Roychoudhury P Dutta TK Subudhi PK Kumar S Gali JM. Isolation and molecular characterization of Gp5 glycoprotein gene of Betaarterivirus Suid 2 from Mizoram, India. Virus. (2021) 32:748–56. doi: 10.1007/s13337-021-00735-x, PMID: 34458505 PMC8378527

[20] Zhou L Yang Y Xia Q Guan Z Zhang J Li B. Genetic characterization of porcine reproductive and respiratory syndrome virus from eastern China during 2017-2022. Front Microbiol. (2022) 13:971817. doi: 10.3389/fmicb.2022.971817, PMID: 36312912 PMC9606797

[21] Li C Fan A Liu Z Wang G Zhou L Zhang H. Prevalence, time of infection, and diversity of porcine reproductive and respiratory syndrome virus in China. Viruses. (2024) 16:774. doi: 10.3390/v16050774, PMID: 38793655 PMC11125865

[22] Mei Y Chen J Chen Y Hu C Chen X Guo A. Porcine reproductive and respiratory syndrome virus prevalence and pathogenicity of one Nadc34-like virus isolate circulating in China. Microorganisms. (2025) 13:796. doi: 10.3390/microorganisms13040796, PMID: 40284632 PMC12029175

[23] Yuan N Yang Z Lv F Dou L Li X Zhao B. Molecular epidemiology and genetic evolution of porcine reproductive and respiratory syndrome virus in northern China during 2021-2023. Viruses. (2025) 17:85. doi: 10.3390/v17010085, PMID: 39861874 PMC11769476

[24] Zhu Z Yuan L Hu D Lian Z Yao X Liu P. Isolation and genomic characterization of a Chinese Nadc34-like Prrsv Isolated from Jiangsu Province. Transbound Emerg Dis. (2022) 69:e1015–27. doi: 10.1111/tbed.14392, PMID: 34786872

[25] Sun S Zhang K Zhang J Zhang P He P Deng D. A novel peptide-based enzyme-linked immunosorbent assay (Elisa) for detection of neutralizing antibodies against Nadc30-like Prrsv Gp5 protein. Int J Mol Sci. (2025) 26:2619. doi: 10.3390/ijms26062619, PMID: 40141266 PMC11941917

[26] Jian Y Lu C Shi Y Kong X Song J Wang J. Genetic evolution analysis of Prrsv Orf5 gene in five provinces of northern China in 2024. BMC Vet Res. (2025) 21:242. doi: 10.1186/s12917-025-04679-y, PMID: 40176022 PMC11966811

[27] Ostrowski M Galeota JA Jar AM Platt KB Osorio FA Lopez OJ. Identification of neutralizing and nonneutralizing epitopes in the porcine reproductive and respiratory syndrome virus Gp5 Ectodomain. J Virol. (2002) 76:4241–50. doi: 10.1128/jvi.76.9.4241-4250.2002, PMID: 11932389 PMC155073

[28] Zhu H Wei L Liu X Liu S Chen H Chen P. Pathogenicity studies of Nadc34-like porcine reproductive and respiratory syndrome virus Lnsy-Gy and Nadc30-like porcine reproductive and respiratory syndrome virus Gxgg-8011 in piglets. Viruses. (2023) 15:2247. doi: 10.3390/v15112247, PMID: 38005924 PMC10674415

[29] Zhou L Yu J Zhou J Long Y Xiao L Fan Y. A novel Nadc34-like porcine reproductive and respiratory syndrome virus 2 with complex genome recombination is highly pathogenic to piglets. Infect Genet Evol. (2023) 112:105436. doi: 10.1016/j.meegid.2023.105436, PMID: 37094706

[30] Rupasinghe R Lee K Liu X Gauger PC Zhang J Martínez-López B. Molecular evolution of porcine reproductive and respiratory syndrome virus field strains from two swine production Systems in the Midwestern United States from 2001 to 2020. Microbiol Spectr. (2022) 10:e0263421. doi: 10.1128/spectrum.02634-21, PMID: 35499352 PMC9241855

[31] Wei Z Lin T Sun L Li Y Wang X Gao F. N-linked glycosylation of Gp5 of porcine reproductive and respiratory syndrome virus is critically important for virus replication in vivo. J Virol. (2012) 86:9941–51. doi: 10.1128/jvi.07067-11, PMID: 22761373 PMC3446624

[32] Luo Q Zheng Y Zhang H Yang Z Sha H Kong W. Research Progress on glycoprotein 5 of porcine reproductive and respiratory syndrome virus. Animals. (2023) 13:813. doi: 10.3390/ani13050813, PMID: 36899670 PMC10000246

[33] Wang PP Dong JG Zhang LY Liang PS Liu YL Wang L. Sequence and phylogenetic analyses of the Nsp2 and Orf5 genes of porcine reproductive and respiratory syndrome virus in boars from South China in 2015. Transbound Emerg Dis. (2017) 64:1953–64. doi: 10.1111/tbed.12594, PMID: 27888586

[34] Xu H Li C Li W Zhao J Gong B Sun Q. Novel characteristics of Chinese Nadc34-like Prrsv during 2020-2021. Transbound Emerg Dis. (2022) 69:e3215–24. doi: 10.1111/tbed.14485, PMID: 35182461

[35] Zhou L Yang X Tian Y Yin S Geng G Ge X. Genetic diversity analysis of genotype 2 porcine reproductive and respiratory syndrome viruses emerging in recent years in China. Biomed Res Int. (2014) 2014:748068. doi: 10.1155/2014/748068, PMID: 24719885 PMC3955690

[36] Jakab S Bali K Freytag C Pataki A Fehér E Halas M. Deep sequencing of porcine reproductive and respiratory syndrome virus Orf7: a promising tool for diagnostics and epidemiologic surveillance. Animals. (2023) 13:3223. doi: 10.3390/ani13203223, PMID: 37893946 PMC10603690

[37] Dortmans J Buter GJ Dijkman R Houben M Duinhof TF. Molecular characterization of type 1 porcine reproductive and respiratory syndrome viruses (Prrsv) isolated in the Netherlands from 2014 to 2016. PLoS One. (2019) 14:e0218481. doi: 10.1371/journal.pone.0218481, PMID: 31246977 PMC6597066

[38] Hao X Lu Z Kuang W Sun P Fu Y Wu L. Polymorphic genetic characterization of the Orf7 gene of porcine reproductive and respiratory syndrome virus (Prrsv) in China. Virol J. (2011) 8:73. doi: 10.1186/1743-422x-8-73, PMID: 21333014 PMC3049123

[39] Kappes MA Faaberg KS. Prrsv structure, replication and recombination: origin of phenotype and genotype diversity. Virology. (2015) 479-480:475–86. doi: 10.1016/j.virol.2015.02.012, PMID: 25759097 PMC7111637

[40] Tian Z Li Q Xu L Liang D Li Y Shi Z. Isolation and pathogenicity of a natural recombinant pig reproductive and respiratory syndrome virus in Northeast China. Viruses. (2025) 17:729. doi: 10.3390/v17050729, PMID: 40431740 PMC12115497

[41] Jeong H Eo Y Lee D Jang G Min KC Choi AK. Comparative genomic and biological investigation of Nadc30- and Nadc34-like Prrsv strains isolated in South Korea. Transbound Emerg Dis. (2025) 2025:9015349. doi: 10.1155/tbed/9015349, PMID: 40302751 PMC12016814

[42] Chen N Ye M Xiao Y Li S Huang Y Li X. Development of universal and Quadruplex real-time Rt-Pcr assays for simultaneous detection and differentiation of porcine reproductive and respiratory syndrome viruses. Transbound Emerg Dis. (2019) 66:2271–8. doi: 10.1111/tbed.13276, PMID: 31233656

[43] Li C Xu H Zhao J Gong B Sun Q Xiang L. Epidemiological investigation and genetic evolutionary analysis of Prrsv-1 on a pig farm in China. Front Microbiol. (2022) 13:1067173. doi: 10.3389/fmicb.2022.1067173, PMID: 36532471 PMC9751794

[44] Yin B Qi S Sha W Qin H Liu L Yun J. Molecular characterization of the Nsp2 and Orf5 (Orf5a) genes of Prrsv strains in nine provinces of China during 2016-2018. Front Vet Sci. (2021) 8:605832. doi: 10.3389/fvets.2021.605832, PMID: 33748205 PMC7969665

