# Supplementary material for: Epidemiology and genetic characterization of porcine reproductive and respiratory syndrome virus in Fujian Province, China, from 2023 to 2024
Source: Front Vet Sci. 2025 Jul 2;12:1634353. doi: 10.3389/fvets.2025.1634353 (PMC12263401; doi:10.3389/fvets.2025.1634353)
Supplement: Supplementary file 1 [file Table_1.DOCX]

Supplementary Table

Table S1 Information on the sources of samples collected from pigs with suspected PRRS disease

| No.  farms | City | Time of collection  （YYMM） | Type and number of collected sample | | | | Vaccination | Clinical health status^b^ |
| --- | --- | --- | --- | --- | --- | --- | --- | --- |
|  |  |  | Serum | Oral fluid | Testicular fluid | Tissue |  |  |
| 1^a^ | Longyan | 202302 | 0 | 0 | 1 | 0 | No | Sick |
| 2 | Fuzhou | 202302 | 9 | 0 | 0 | 0 | No | Normal |
| 3 | Ningde | 202302 | 0 | 4 | 0 | 0 | No | Sick |
| 4 | Quanzhou | 202302 | 0 | 0 | 1 | 0 | Yes | Sick |
| 5 | Longyan | 202302 | 0 | 6 | 0 | 0 | Yes | Normal |
| 6 | Nanping | 202303 | 0 | 0 | 0 | 5 | No | Sick |
| 7 | Quanzhou | 202303 | 4 | 0 | 0 | 0 | Yes | Sick |
| 8 | Nanping | 202303 | 0 | 0 | 0 | 4 | No | Normal |
| 9 | Zhangzhou | 202303 | 0 | 0 | 1 | 0 | No | Normal |
| 10 | Putian | 202304 | 4 | 0 | 0 | 0 | Yes | Normal |
| 11 | Longyan | 202304 | 4 | 0 | 0 | 0 | No | Sick |
| 12 | Quanzhou | 202304 | 0 | 0 | 0 | 2 | Yes | Normal |
| 13 | Nanping | 202304 | 0 | 0 | 1 | 0 | No | Sick |
| 14 | Longyan | 202304 | 9 | 0 | 0 | 0 | Yes | Normal |
| 15 | Fuzhou | 202304 | 0 | 0 | 0 | 5 | No | Sick |
| 16 | Quanzhou | 202305 | 5 | 0 | 0 | 0 | No | Normal |
| 17 | Sanming | 202305 | 0 | 0 | 0 | 3 | Yes | Sick |
| 18 | Longyan | 202305 | 0 | 8 | 0 | 0 | Yes | Normal |
| 19 | Ningde | 202305 | 0 | 0 | 0 | 1 | Yes | Sick |
| 20 | Putian | 202305 | 0 | 0 | 0 | 3 | No | Normal |
| 21 | Fuzhou | 202305 | 0 | 0 | 0 | 1 | Yes | Sick |
| 22 | Longyan | 202305 | 0 | 0 | 0 | 2 | Yes | Normal |
| 23 | Longyan | 202305 | 0 | 0 | 0 | 1 | No | Normal |
| 24 | Sanming | 202305 | 0 | 0 | 0 | 1 | No | Sick |
| 25 | Nanping | 202306 | 0 | 0 | 0 | 1 | No | Sick |
| 26 | Putian | 202306 | 0 | 3 | 0 | 0 | Yes | Normal |
| 27 | Longyan | 202306 | 0 | 0 | 0 | 1 | Yes | Normal |
| 28 | Ningde | 202306 | 0 | 0 | 0 | 3 | Yes | Normal |
| 29 | Nanping | 202307 | 0 | 0 | 0 | 3 | Yes | Normal |
| 30 | Sanming | 202307 | 0 | 0 | 0 | 2 | No | Normal |
| 31 | Ningde | 202307 | 0 | 0 | 0 | 1 | No | Normal |
| 32 | Zhangzhou | 202307 | 2 | 0 | 0 | 0 | No | Sick |
| 33 | Quanzhou | 202307 | 0 | 0 | 0 | 3 | No | Normal |
| 34 | Zhangzhou | 202307 | 0 | 0 | 0 | 3 | Yes | Normal |
| 35 | Nanping | 202308 | 0 | 0 | 0 | 2 | No | Sick |
| 36 | Longyan | 202308 | 0 | 0 | 0 | 0 | Yes | Sick |
| 37 | Nanping | 202308 | 9 | 0 | 0 | 0 | No | Normal |
| 38 | Zhangzhou | 202308 | 0 | 0 | 0 | 3 | Yes | Normal |
| 39 | Fuzhou | 202308 | 0 | 0 | 0 | 0 | No | Sick |
| 40 | Quanzhou | 202308 | 0 | 0 | 0 | 5 | Yes | Normal |
| 41 | Nanping | 202308 | 0 | 0 | 0 | 1 | Yes | Normal |
| 42 | Fuzhou | 202308 | 0 | 0 | 0 | 1 | Yes | Sick |
| 43 | Quanzhou | 202308 | 0 | 0 | 0 | 2 | No | Sick |
| 44 | Putian | 202309 | 0 | 0 | 0 | 1 | Yes | Normal |
| 45 | Sanming | 202309 | 0 | 0 | 13 | 0 | No | Normal |
| 46 | Ningde | 202309 | 0 | 0 | 0 | 0 | Yes | Normal |
| 47 | Putian | 202309 | 0 | 0 | 0 | 3 | Yes | Sick |
| 48 | Nanping | 202309 | 0 | 0 | 0 | 1 | Yes | Normal |
| 49 | Ningde | 202310 | 0 | 0 | 0 | 1 | No | Sick |
| 50 | Zhangzhou | 202310 | 0 | 0 | 0 | 2 | No | Sick |
| 51 | Putian | 202311 | 0 | 0 | 0 | 1 | Yes | Sick |
| 52 | Zhangzhou | 202311 | 0 | 13 | 0 | 0 | Yes | Normal |
| 53 | Quanzhou | 202311 | 0 | 0 | 0 | 4 | No | Normal |
| 54 | Putian | 202311 | 0 | 0 | 0 | 2 | Yes | Normal |
| 55 | Ningde | 202311 | 0 | 0 | 0 | 1 | Yes | Sick |
| 56 | Zhangzhou | 202312 | 0 | 0 | 0 | 2 | No | Sick |
| 57 | Longyan | 202312 | 0 | 0 | 0 | 2 | Yes | Sick |
| 58 | Zhangzhou | 202312 | 0 | 0 | 0 | 2 | Yes | Normal |
| 59 | Longyan | 202312 | 0 | 0 | 0 | 5 | No | Normal |
| 60 | Zhangzhou | 202401 | 0 | 0 | 0 | 4 | Yes | Normal |
| 61 | Sanming | 202401 | 0 | 0 | 0 | 3 | Yes | Normal |
| 62 | Quanzhou | 202401 | 0 | 0 | 0 | 1 | Yes | Sick |
| 63 | Quanzhou | 202401 | 0 | 0 | 0 | 1 | No | Normal |
| 64 | Sanming | 202402 | 0 | 0 | 0 | 1 | Yes | Normal |
| 65 | Fuzhou | 202403 | 0 | 0 | 0 | 2 | No | Sick |
| 66 | Fuzhou | 202403 | 0 | 0 | 0 | 1 | Yes | Sick |
| 67 | Fuzhou | 202403 | 0 | 0 | 0 | 1 | No | Normal |
| 68 | Fuzhou | 202403 | 0 | 0 | 0 | 1 | Yes | Sick |
| 69 | Zhangzhou | 202403 | 0 | 0 | 0 | 3 | Yes | Normal |
| 70 | Longyan | 202403 | 0 | 0 | 0 | 3 | No | Normal |
| 71 | Sanming | 202403 | 0 | 0 | 0 | 1 | Yes | Normal |
| 72 | Ningde | 202403 | 0 | 0 | 12 | 0 | No | Normal |
| 73 | Sanming | 202404 | 0 | 0 | 0 | 1 | Yes | Sick |
| 74 | Sanming | 202404 | 0 | 0 | 0 | 3 | Yes | Normal |
| 75 | Fuzhou | 202404 | 0 | 5 | 0 | 0 | No | Normal |
| 76 | Nanping | 202404 | 0 | 0 | 0 | 3 | No | Sick |
| 77 | Ningde | 202404 | 5 | 0 | 0 | 0 | Yes | Normal |
| 78 | Ningde | 202404 | 0 | 0 | 0 | 3 | No | Sick |
| 79 | Putian | 202404 | 3 | 0 | 0 | 0 | No | Normal |
| 80 | Quanzhou | 202404 | 0 | 8 | 0 | 0 | Yes | Normal |
| 81 | Zhangzhou | 202404 | 0 | 0 | 0 | 1 | No | Sick |
| 82 | Nanping | 202404 | 0 | 0 | 0 | 2 | Yes | Normal |
| 83 | Nanping | 202405 | 0 | 0 | 0 | 1 | Yes | Sick |
| 84 | Longyan | 202405 | 0 | 0 | 0 | 1 | No | Normal |
| 85 | Ningde | 202405 | 0 | 0 | 0 | 5 | Yes | Sick |
| 86 | Sanming | 202406 | 3 | 0 | 0 | 0 | No | Sick |
| 87 | Longyan | 202406 | 0 | 0 | 0 | 1 | Yes | Sick |

^a^ The farm marked in red indicates that at least one sample collected from it tested positive for PRRSV.

^b^ “Sick” or “Normal” denotes whether the sampled herd is determined to be diseased or healthy status based on veterinary clinical observation. The clinical signs in question are those relevant to PRRSV, including fever, coughing, abortion, and inappetence.

Table S2 The reference strains used in this study.

| Strain name | Isolated area | Isolated time | GenBank accession | Genotype* |
| --- | --- | --- | --- | --- |
| LNWK130 | China | 2017 | MG913987 | Sublineage 1.5 |
| FJ0908 | China | 2018 | MK202794 | Sublineage 1.5 |
| NADC34 | USA | 2014 | MF326985 | Sublineage 1.5 |
| NADC30 | USA | 2008 | JN654459 | Sublineage 1.8 |
| CHsx1401 | China | 2014 | KP861625 | Sublineage 1.8 |
| HeN1 | China | 2015 | KX815413 | Sublineage 1.8 |
| QYYZ | China | 2011 | JQ308798 | Lineage 3 |
| GM2 | China | 2011 | JN662424 | Lineage 3 |
| GDKP | China | 2015 | KU978619 | Lineage 3 |
| VR2332 | USA | 1995 | U87392 | Lineage 5 |
| S1 | China | 1998 | DQ459471 | Lineage 5 |
| RespPRRS MLV | USA | 1994 | AF066183 | Lineage 5 |
| BJ-4 | China | 1996 | AF331831 | Lineage 5 |
| TJ | China | 2006 | EU860248 | Lineage 8 |
| JXA1 | China | 2006 | EF112445 | Lineage 8 |
| CH-1a | China | 1996 | AY032626 | Lineage 8 |
| Lelystad virus | Netherlands | 1993 | M96262 | PRRSV-1 |

* Classification framework based on the PRRSV ORF5 gene

Table S3 Information of 37 PRRSV isolates from Fujian China in this study.

| Strain name | Province（state） | Gene | Accession | Collection-date |
| --- | --- | --- | --- | --- |
| FJLY2302 | Fujian（Longyan） | NSP2 | PQ465885 | Feb-2023 |
|  |  | ORF5 | PQ465923 |  |
|  |  | ORF7 | PQ465960 |  |
| FJQZ2302 | Fujian（Quanzhou） | NSP2 | PQ465886 | Feb-2023 |
|  |  | ORF5 | PQ465924 |  |
|  |  | ORF7 | PQ465961 |  |
| FJZZ2303 | Fujian（Zhangzhou） | NSP2 | PQ465887 | Mar-2023 |
|  |  | ORF5 | PQ465925 |  |
|  |  | ORF7 | PQ465962 |  |
| FJNP2304 | Fujian（Nanping） | NSP2 | PQ465888 | Apr-2023 |
|  |  | ORF5 | PQ465926 |  |
|  |  | ORF7 | PQ465963 |  |
| FJND2305 | Fujian（Ningde） | NSP2 | PQ465889 | May-2023 |
|  |  | ORF5 | PQ465927 |  |
|  |  | ORF7 | PQ465964 |  |
| FJSM2305 | Fujian（Sanming） | NSP2 | PQ465890 | May-2023 |
|  |  | ORF5 | PQ465928 |  |
|  |  | ORF7 | PQ465965 |  |
| FJFZ2305 | Fujian（Fuzhou） | NSP2 | PQ465891 | May-2023 |
|  |  | ORF5 | PQ465929 |  |
|  |  | ORF7 | PQ465966 |  |
| FJLY2305 | Fujian（Longyan） | NSP2 | PQ465892 | May-2023 |
|  |  | ORF5 | PQ465930 |  |
|  |  | ORF7 | PQ465967 |  |
| FJLY2306 | Fujian（Longyan） | NSP2 | PQ465893 | Jun-2023 |
|  |  | ORF5 | PQ465931 |  |
|  |  | ORF7 | PQ465968 |  |
| FJNP2306 | Fujian（Nanping） | NSP2 | PQ465894 | Jun-2023 |
|  |  | ORF5 | PQ465932 |  |
|  |  | ORF7 | PQ465969 |  |
| FJND2307 | Fujian（Ningde） | NSP2 | PQ465895 | Jul-2023 |
|  |  | ORF5 | PQ465933 |  |
|  |  | ORF7 | PQ465970 |  |
| FJZZ2307 | Fujian（Zhangzhou） | NSP2 | PQ465896 | Jul-2023 |
|  |  | ORF5 | PQ465934 |  |
|  |  | ORF7 | PQ465971 |  |
| FJFZ2308 | Fujian（Fuzhou） | NSP2 | PQ465897 | Aug-2023 |
|  |  | ORF5 | PQ465935 |  |
|  |  | ORF7 | PQ465972 |  |
| FJQZ2308 | Fujian（Quanzhou） | NSP2 | PQ465898 | Aug-2023 |
|  |  | ORF5 | PQ465936 |  |
|  |  | ORF7 | PQ465973 |  |
| FJNP2308 | Fujian（Nanping） | NSP2 | PQ465899 | Aug-2023 |
|  |  | ORF5 | PQ465937 |  |
|  |  | ORF7 | PQ465974 |  |
| FJPT2309 | Fujian（Putian） | NSP2 | PQ465900 | Sep-2023 |
|  |  | ORF5 | PQ465938 |  |
|  |  | ORF7 | PQ465975 |  |
| FJNP2309 | Fujian（Nanping） | NSP2 | PQ465901 | Sep-2023 |
|  |  | ORF5 | PQ465939 |  |
|  |  | ORF7 | PQ465976 |  |
| FJND2310 | Fujian（Ningde） | NSP2 | PQ465902 | Oct-2023 |
|  |  | ORF5 | PQ465940 |  |
|  |  | ORF7 | PQ465977 |  |
| FJZZ2310 | Fujian（Zhangzhou） | NSP2 | PQ465903 | Oct-2023 |
|  |  | ORF5 | PQ465941 |  |
|  |  | ORF7 | PQ465978 |  |
| FJPT2311 | Fujian（Putian） | NSP2 | PQ465904 | Nov-2023 |
|  |  | ORF5 | PQ465942 |  |
|  |  | ORF7 | PQ465979 |  |
| FJND2311 | Fujian（Ningde） | NSP2 | PQ465905 | Nov-2023 |
|  |  | ORF5 | PQ465943 |  |
|  |  | ORF7 | PQ465980 |  |
| FJZZ2312 | Fujian（Zhangzhou） | NSP2 | PQ465906 | Dec-2023 |
|  |  | ORF5 | PQ465944 |  |
|  |  | ORF7 | PQ465981 |  |
| FJLY2312(1) | Fujian（Longyan） | NSP2 | PQ465907 | Dec-2023 |
|  |  | ORF5 | PQ465945 |  |
|  |  | ORF7 | PQ465982 |  |
| FJLY2312(2) | Fujian（Longyan） | NSP2 | PQ465908 | Dec-2023 |
|  |  | ORF5 | PQ465945 |  |
|  |  | ORF7 | PQ465982 |  |
| FJSM2402 | Fujian（Sanming） | NSP2 | PQ465909 | Feb-2024 |
|  |  | ORF5 | PQ465946 |  |
|  |  | ORF7 | PQ465983 |  |
| FJFZ2403-1 | Fujian（Fuzhou） | NSP2 | PQ465910 | Mar-2024 |
|  |  | ORF5 | PQ465947 |  |
|  |  | ORF7 | PQ465984 |  |
| FJFZ2403-2 | Fujian（Fuzhou） | NSP2 | PQ465911 | Mar-2024 |
|  |  | ORF5 | PQ465948 |  |
|  |  | ORF7 | PQ465985 |  |
| FJFZ2403-3 | Fujian（Fuzhou） | NSP2 | PQ465912 | Mar-2024 |
|  |  | ORF5 | PQ465949 |  |
|  |  | ORF7 | PQ465986 |  |
| FJFZ2403-4 | Fujian（Fuzhou） | NSP2 | PQ465913 | Mar-2024 |
|  |  | ORF5 | PQ465950 |  |
|  |  | ORF7 | PQ465987 |  |
| FJSM2403 | Fujian（Sanming） | NSP2 | PQ465914 | Mar-2024 |
|  |  | ORF5 | PQ465951 |  |
|  |  | ORF7 | PQ465988 |  |
| FJQZ2404-1 | Fujian（Quazhou） | NSP2 | PQ465915 | Apr-2024 |
|  |  | ORF5 | PQ465952 |  |
|  |  | ORF7 | PQ465989 |  |
| FJQZ2404-2 | Fujian（Quazhou） | NSP2 | PQ465916 | Apr-2024 |
|  |  | ORF5 | PQ465953 |  |
|  |  | ORF7 | PQ465990 |  |
| FJSM2404 | Fujian（Sanming） | NSP2 | PQ465917 | Apr-2024 |
|  |  | ORF5 | PQ465954 |  |
|  |  | ORF7 | PQ465991 |  |
| FJZZ2404 | Fujian（Zhangzhou） | NSP2 | PQ465918 | Apr-2024 |
|  |  | ORF5 | PQ465955 |  |
|  |  | ORF7 | PQ465992 |  |
| FJNP2404 | Fujian（Nanping） | NSP2 | PQ465919 | Apr-2024 |
|  |  | ORF5 | PQ465956 |  |
|  |  | ORF7 | PQ465993 |  |
| FJNP2405 | Fujian（Nanping） | NSP2 | PQ465920 | May-2024 |
|  |  | ORF5 | PQ465957 |  |
|  |  | ORF7 | PQ465994 |  |
| FJLY2405 | Fujian（Longyan） | NSP2 | PQ465921 | May-2024 |
|  |  | ORF5 | PQ465958 |  |
|  |  | ORF7 | PQ465995 |  |
| FJND2406 | Fujian（Ningde） | NSP2 | PQ465922 | Jun-2024 |
|  |  | ORF5 | PQ465959 |  |
|  |  | ORF7 | PQ465996 |  |

Table S4 Critical amino acid substitutions in GP5 function epitopes of various PRRSV-2 genotypes.

| Functional epitopes | Position | PRRSV-2 genotypes | | | | |
| --- | --- | --- | --- | --- | --- | --- |
|  |  | Sublineage 1.5 | Sublineage 1.8 | Lineage 3 | Lineage 5 | Lineage 8 |
| Signal Peptide | 3 | G | G | G | E | G |
|  | 13 | R/Q | Q/H/S/R | Q/P | Q/R | R |
|  | 16 | F | F/S | F | S | F/S |
|  | 25 | F/L | L/F/P | S | F | L/F |
|  | 26 | V | A/V | I | A | A/V |
|  | 27 | A | A/V | A | V/A | V |
|  | 29 | V/A | V/A | V/A | A | V |
|  | 32 | S/N | S/N | S/N/D | S | S/N |
| Primary neutralizing epitope | 38 | H | H | Y | H | H/R |
|  | 39 | L | L | S | L | I/F |
| B cell epitopes | 3 | G | G | G | E | G |
|  | 29 | V/A | V/A | V/A | A | V |
|  | 32 | S/N | S/N | S/N/D | S | S/N |
|  | 34 | S/T/N | N/H/D/S | N | D | N/S |
|  | 38 | H | H | Y | H | H/R |
|  | 39 | L | L | S | L | I/F |
|  | 152 | L | L | I | L | L |
|  | 168 | E | D | E | E | E |
| T cell epitopes | 32 | S/N | S/N | S/N/D | S | S/N |
|  | 34 | S/T/N | N/H/D/S | N | D | N/S |
|  | 117 | L | L | F | L | L |
|  | 121 | T | I/V/A | V/I | T | V/I |
|  | 126 | R | S/R | R | F | R |
|  | 128 | T | A/V | A/V | A | T/A |
| NGSs | 32 | S/N | S/N | S/N/D | S | S/N |
|  | 34 | S/T/N | N/H/D/S | N | D | N/S |
| Transmembrane domain | 66 | T | T | C | S | T |
|  | 92 | A/G | G | S/T | A | G |
|  | 94 | I | V/A/L/I | V | V | A/V |
|  | 101 | Y | Y | F/Y | F | F/Y |
|  | 102 | Y/C | Y/C/H | Y/C/H/R | V | C |
|  | 117 | L | L | F | L | L |
|  | 121 | T | I/V/A | V/I | T | V/I |
|  | 126 | R | S/R | R | F | R |
|  | 128 | T | A/V | A/V | A | T/A |
